# Gut health benefit and application of postbiotics in animal production

**DOI:** 10.1186/s40104-022-00688-1

**Published:** 2022-04-08

**Authors:** Yifan Zhong, Shanshan Wang, Hanqiu Di, Zhaoxi Deng, Jianxin Liu, Haifeng Wang

**Affiliations:** College of Animal Science, Zhejiang University, The Key Laboratory of Molecular Animal Nutrition, Ministry of Education, Hangzhou, 310058 China

**Keywords:** Animal production, Gut health, Postbiotics

## Abstract

Gut homeostasis is of importance to host health and imbalance of the gut usually leads to disorders or diseases for both human and animal. Postbiotics have been applied in manipulating of gut health, and utilization of postbiotics threads new lights into the host health. Compared with the application of probiotics, the characteristics such as stability and safety of postbiotics make it a potential alternative to probiotics. Studies have reported the beneficial effects of components derived from postbiotics, mainly through the mechanisms including inhibition of pathogens, strengthen gut barrier, and/or regulation of immunity of the host. In this review, we summarized the characteristics of postbiotics, main compounds of postbiotics, potential mechanisms in gut health, and their application in animal production.

## Introduction

Gut homeostasis has been demonstrated to be with importance in maintaining human and animal health [1, 2], and there is mounting evidence that gut microbiota plays a vital role in this function [3, 4]. Although it remains challenging, modulation of complex interactions between gut microbes and host health shows a promise in growth [5], fertility [6], aging [7], disease [8]. It is well established that supplementary probiotics can benefit the host, including specific strains from *Lactobacillus* [9], *Bifidobacterium* [10], and *Akkermansia* [11]. The term of probiotics, “Live microorganisms which when administered in adequate amounts, confer a health benefit on the host,” [12] has been widely accepted. The probiotics improve host health via supporting a healthy digestive tract and/or a healthy immune system [13], mainly through producing useful metabolites or enzymes [14, 15]. Since probiotics were defined as live microorganisms and probiotic products have been widely applied, large numbers of dead and injured microorganisms existed [16, 17], still maintaining the influence on host health while having little attention. The beneficial effects of components and end-products from non-viable microorganisms were also observed, such as bacterial lysates [18], lactic acid [19], short-chain fatty acids (SCFAs) [20], bioactive peptides [21]. Moreover, appropriate applications of probiotics remain uncertain because they are alive when administered. The safety of probiotics [22, 23] and complex interactions between gut microbiota [24] have not been totally illustrated yet. Postbiotics were proposed and bring new inspiration for the modulation of gut health due to their advantages. Here, we provided a review of the postbiotics, including their definition, potential mechanisms, and application in animal production.

### Postbiotics and its advantages in utilization

Several terms of postbiotics have existed and used, for example, ‘Tyndallized probiotics’ [25], ‘Heat-killed probiotics’ [26], ‘Paraprobiotics’ [27], and ‘Bacterial lysates’ [28]. Although studies and publications of “postbiotics” are increasing steadily [29], the precise definition of “postbiotics” remains under discussion [30]. The term of “postbiotics” was first coined by Tsilingiri et al., which are metabolic products derived from probiotics that exert beneficial effects on the host via direct or indirect way [31, 32]. In 2019, definition of ‘postbiotic’ was proposed as “preparation of inanimate microorganisms and/or their components that confers a health benefit on the host” by International Scientific Association of Probiotics and Prebiotics (ISAPP) [33].

The safety of probiotics is associated with their further utilization. Although few studies have reported on this issue, potential risks of probiotics existed, including genetic stability, infectivity, or in situ toxin production [34, 35]. Postbiotics are inanimate microorganisms or their product those lose the capacity to replicate or produce and are free from the concerns above. However, a lower risk of postbiotics does not mean that there is no risk. Specific toxic metabolites or substrates might be released from dead bacteria [36], which still need to be further assessed.

The rate of live microorganisms in probiotics is uncertain at the end of shelf life due to the death of live microorganisms during different storage conditions [37]. Therefore, probiotics is commonly included in excess of dose to avoid the loss of live microorganisms during the production [38]. In contrast, the potential effects of dead microorganisms in the probiotic products were usually ignored. Postbiotics can maintain stability during industrial process and storage in long shelf life, making it more potentialities in application than probiotics [29]. Thus, it is with possibility to control the precision amount of postbiotics in the products during processing.

### Components of postbiotics

**Fig. 1:**
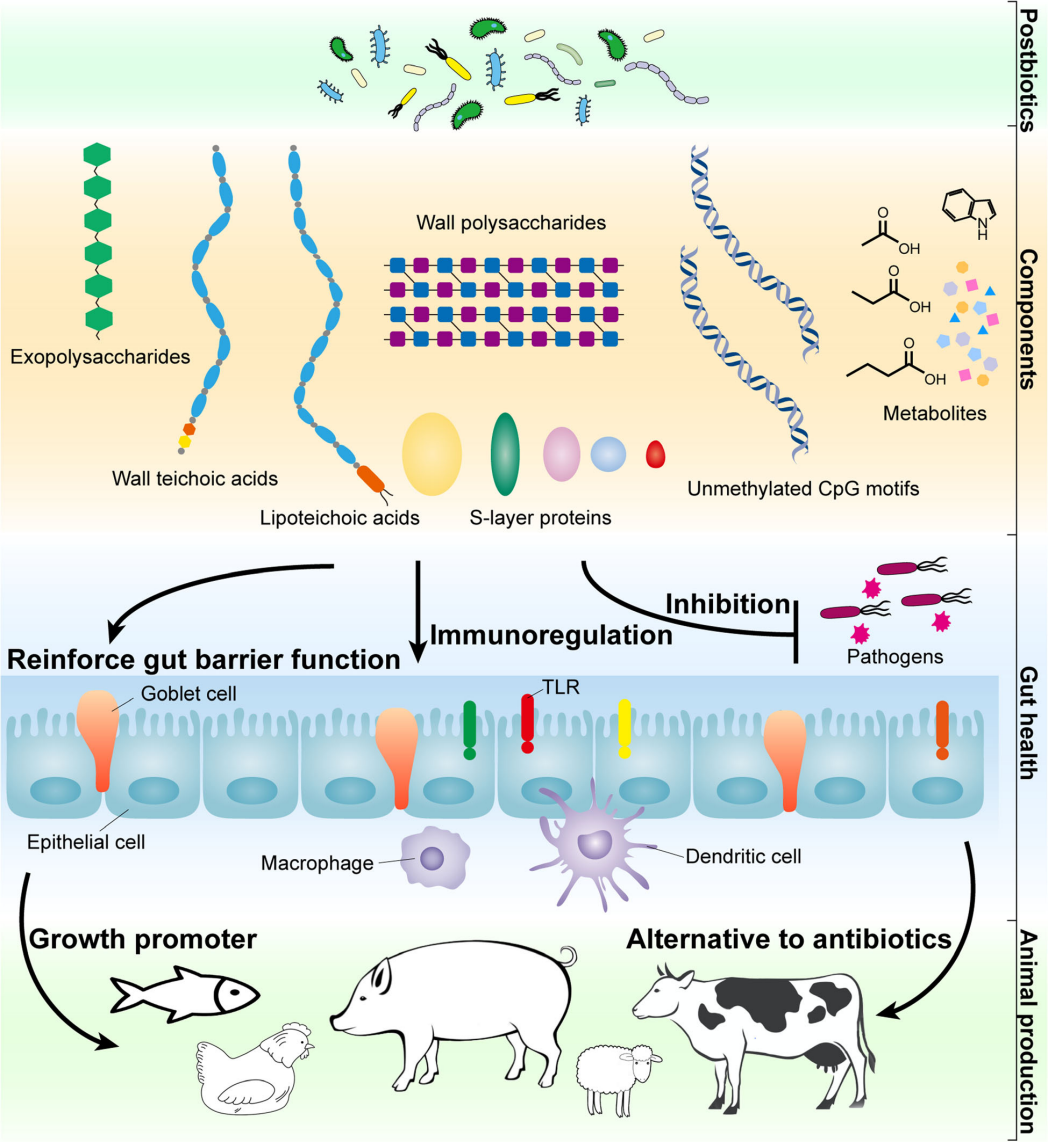
Beneficial compounds and potential mechanisms of postbiotics in gut health and animal production. The components of postbiotics, including exopolysaccharides, wall teichoic acids, lipoteichoic acids, wall polysaccharides, S-layer proteins, unmethylated CpG motifs, metabolites exert beneficial effects on the gut health, mainly through the inhibition of pathogens, reinforce gut barrier function, and immunoregulation mechanisms. Postbiotics can be used as growth promoter and alternative to antibiotics in animal production. CpG, cytosine-guanine dinucleotide in particular base contexts; TLR, Toll-like receptor

Diverse components and molecules derived from microorganisms still exist in postbiotics after processing, contributing to host health in different ways. To discover the beneficial effects and mechanisms of components in postbiotics, they were purified and administrated in both in vivo and in vitro studies. In this part, we summarize the potentially probiotic components as postbiotics reported in previous studies, and these components includes exopolysaccharides, wall polysaccharides, teichoic acids (wall teichoic acids and lipoteichoic acids), surface layer proteins and bacterial DNA and metabolites and so on (Fig. 1).

#### Exopolysaccharides

Exopolysaccharides (EPS) are extracellular carbohydrate polymers with high molecular weight compounds produced and secreted by microorganisms [39], which attracted attention due to the therapeutic potential in medical applications and the food industry during the past decades [40]. EPS can be found abundantly in lactic acid bacteria (LAB), including *Lactobacillus*, *Lactococcus*, *Bifidobacterium*, *Leuconostoc*, *Pediococcus*, *Streptococcus*, and *Weissella* [41, 42]. EPS such as xanthan, sphingan, alginate, cellulose show the capability in water-binding, water-retention water, and immense swelling and gelation, which could act as a protective barrier via promoting biofilm formation on the bacterial cell surfaces [43, 44]. Beneficial effects of EPS to gut health were observed, including antimicrobial [45], immunomodulatory [46], and anti-inflammatory activities [47]. An in vitro study revealed the EPS produced by *Lactobacillus rhamnosus* isolated from human breast milk showed substantial antibacterial activity against the pathogens *Salmonella enterica* serovar Typhimurium and *Escherichia coli* [48]. The previous study showed pretreatment of IPEC-J2 cells with EPS isolated from *L. rhamnosus* GG (LGG) could attenuate LPS-induced MAPK and NF-κB as well as alleviate the inflammatory cytokines and TLR activation at mRNA level [49]. Moreover, EPS could prevent bacterial adhesion to the epithelium and contribute to the epithelial barrier integrity in the gut [50]. An in vivo studies showed EPS derived from *Bifidobacterium breve* UCC2003 could prevent bacterial adhesion to the intestinal epithelium [51]. Transepithelial electrical resistance (TEER) is often used to assess epithelial cell barrier function [52]. Exopolysaccharides from *Lactobacillus plantarum* NCU116 induce apoptosis via TLR2 in mouse intestinal epithelial cancer cells, which demonstrated that EPS116/TLR2/MyD88 signaling activated c-Jun N-terminal kinase (JNK) and promoted c-Jun phosphorylation to promote upregulation of Fas/Fasl and to trigger apoptotic signaling [53].

#### Cellular wall fragments

Most of the probiotics to date, including *Lactobacillus* and *Bifidobacterium*, are gram-positive [54]. The cell wall of gram-positive bacteria is a complex assemblage of peptidoglycan, teichoic acids, polysaccharides, and proteins [55] which are considered beneficial components to the host.

Peptidoglycan consists of β-1,4-linked N-acetylglucosamine and N-acetylmuramic disaccharide units and accounts for approximately 90% of the weight of the cell wall in gram-positive bacteria [56]. Previous studies have revealed that peptidoglycan derived from probiotics or commensal LAB might play a positive role in maintaining the immune balance of the gut. The peptidoglycan of heat-killed *L. casei*, *L. johnsonii* JCM 2012^T^, and *L. plantarum* ATCC 14917^T^ could inhibit the production of IL-12 through Toll-like receptor 2 (TLR2) in the gut, which further maintains homeostasis in the host [57]. The protective capacity of purified peptidoglycan from *L. salivarius* Ls33 was observed in IL-10 dependent pathway through induction of regulatory CD103^+^ DCs and regulatory T cells in the gut of mice [58].

Teichoic acids are anionic polymers made of alditol-phosphate repeating units and can be classified into wall teichoic acids (WTAs) and lipoteichoic acids (LTAs) [59]. The function of teichoic acids in regulation of cell physiology remains to be investigated, but the importance of teichoic acids has been highlighted in host-cell adhesion, inflammation, and immune activation [60]. The immunostimulatory effects of LTAs were observed via binding to TLR2 and activating cytokine release [61]. LTAs purified from *L. casei* YIT 9029 and *L. fermentum* YIT 0159 could induce TNF-α secretion from murine macrophages via a TLR2-mediated strain-dependent mechanism [62]. The structures of WTA are more diverse than those of LTA, and the immune signaling of WTA is still debated [63, 64]. A previous study showed that the purified WTAs of *L. plantarum* strains did not induce the secretion of any cytokines when applied in human dendritic cells [65]. Apart from the beneficial effects of teichoic acid, the safety of teichoic acid still needs to be tested since the excessive inflammatory response might be triggered [30].

Bacterial wall polysaccharides can be divided into three groups, including exopolysaccharides (EPS), capsular polysaccharides (CPS), and cell wall polysaccharides (WPS). Unlike EPSs loosely associated with the cell surface, the CPSs are permanently attached to the cell, and WPS may or may not be covalently attached to the cell wall but do not form a capsule [55]. Since EPS has been discussed above, the beneficial effects of CPS and WPS were discussed here. CPS is a highly hydrated molecule that contains over 95% water, protecting cells from desiccation in adverse conditions [66]. In addition, the CPS was considered to be immunomodulating molecules and has been reported to be important virulence factors in pathogenic bacteria [67, 68]. For LAB, WPS plays a role in cell division and morphology, protection against phagocytosis [69], adhesion, and biofilm formation in bacterial physiology [70], while the beneficial effects of purified WPS remain to be investigated in the future.

The proteinaceous surface layer (S-layer) are the basic components of gram-positive and gram-negative bacteria and provide important functional properties [71]. Proteins in S-layer, known as S-layer proteins (SLPs), represent one of the most abundant cellular proteins and interact with the host and its immune system [72]. The SLPs of probiotics could contribute to the adhesion to epithelial cells and extracellular matrix proteins, thus inhibiting the pathogens’ infections and further benefiting the host [73]. Indeed, SLPs in *Lactobacillus* strains isolated from pig intestine play an important role in adhesion and competitive exclusion of *E. coli* and *Salmonella enteritidis* in Caco-2 cells [74]. Spent culture supernatants of *L. kefir* with significant amounts of SLPs could inhibition the invasion of *Salmonella* in Caco-2/TC-7 cells [75]. SLPs isolated from *L. acidophilus* could block the viral infection via binding DC-specific intercellular adhesion molecule 3-grabbing non-integrin in 3 T3 cells [76].

#### Bacterial DNA

The bacterial DNA can be recognized by the vertebrate immune system, especially unmethylated cytosine-guanine dinucleotide (CpG motifs) in particular base contexts [77]. Unmethylated CpG motifs are prevalent in bacterial but are heavily suppressed and methylated in vertebrate genomic DNAs, which could play an immunomodulatory effect via the TLR9-MyD88-NF-κB signaling pathway [78]. For example, a high frequency of CpG motifs was identified in the DNA of *B. longum* NCC2705, which might be one of the reasons that they play an important role in the immunostimulatory properties [79]. The synthetic oligodeoxynucleotides (ONDs) contain CpG motifs were found to be effective immunotherapy in several diseases, including the treatment of kidney, skin, breast, uterine, and immune malignancies [80]. Furthermore, CpG-ONDs derived from LGG could attenuate inflammatory cytokine TNF-α and IL-6 production in LPS-stimulated cells, which exerted an anti-inflammation effect on epithelial cells [81]. Apart from the unmethylated GpG motifs, the probiotic DNA was also found to possess immune modulation effects. Purified genomic DNA from the mixture of LGG and *B. longum* BB536 could enhance the intestinal barrier function and preventing food allergic response in rats [82]. Moreover, pure DNA of *Bifidobacterium*, which was isolated from feces, also showed an anti-inflammatory effect in peripheral blood mononuclear cells, including the decrease of IL-1β and increase of IL-10 [83].

#### Metabolites

Probiotics could interact with the host via metabolites, including indole, SCFAs, vitamins, and other metabolites. Cell-free supernatants (CFS) contain metabolites derived from probiotics were investigated in several previous studies. CFS of *L. reuteri* AN417, the strain isolated from porcine small intestine, showed greater antimicrobial activity against oral pathogenic bacteria than other *Lactobacillus* strains such as KCTC 3594 and KETC 3678. The carbohydrates and/or fatty acid metabolites in the CFS of *L. reuteri* AN417 might be the main antimicrobial factors in reducing biofilm’s integrity and suppressing the expression of genes involved in biofilm formation [84]. Previously study showed that culture supernatant from probiotics isolated from breast milk-fed infants, including *L. paracasei* CNCM I-4034, *B. breve* CNCM I-4035, and *L. rhamnosus* CNCM I-4036 inhibits the growth of enterotoxigenic and enteropathogenic bacteria [85]. *L. reuteri* ZJ617 isolated from piglets showed probiotic attributes [74], ZJ617 culture supernatant attenuated liver injury induced by LPS via suppression of hepatic TLR4/MAPK/NF-κB activation, apoptosis, and autophagy in mice [86]. The culture supernatant of *L. paracasei* CNCM I-4034 could modulate the Salmonella-induced inflammation of human intestinal-like dendritic and Caco-2 cells [87]. CFS of cultures originated from sixteen strains of Lactobacilli and Bifidobacteria prevented *E. coli* from entering into small and large intestine in human colonic adenocarcinoma cell lines, T84 and Caco2 cells [88].

### Beneficial of postbiotics on gut health

#### Protective effects against pathogens

Disturbance of gut microbiota, such as the colonization of pathogens and overgrowth of indigenous pathobionts, leads to the damage of gut health and diseases. Postbiotics can be used as a therapeutic approach to inhibit pathogens mainly via the components and competition for adhesion to mucosa and epithelium in the gut [89]. Metabolites such as lactic acids, bacteriocins, and SCFAs in postbiotics were observed to have a role in protecting from invasion by pathogens via diffusion across the bacterial membrane and reducing pH value in the gut [90]. Studies showed lactic acid and bacteriocins from lactic acid bacteria have antimicrobial activity and might be the alternatives to antibiotics [91, 92]. Moreover, extensive studies revealed the beneficial effects of SCFAs against the pathogens in the gut, including acetate, propionate, butyrate. Acetate derived in the gut could protect against respiratory syncytial virus infection via activation of GPR43 in pulmonary epithelial cells and promotion IFN-β response in mice [93]. An in vitro study revealed that propionate directly inhibited *S. typhimurium* growth by disrupting intracellular pH homeostasis and mediated the colonization resistance to *S. typhimurium* infection in the gut [94]. Single-cell RNA-sequencing showed the butyrate could imprints potent antimicrobial activity in macrophage differentiation through HDAC3 function [95]. Bacteriocins are small antimicrobial peptides that exhibit inhibitory activity against pathogens and can be a potential candidate for antimicrobial agents in the application of food and pharmaceutics [96, 97]. For example, a purified bacteriocin from *L. helveticus* PJ4 isolated from Wistar Rat showed a bactericidal mode of action against *E. coli* and *E. faecalis* DT48 [98]. The metabolites in postbiotics inhibit pathogens directly but also contribute to cross-feeding on micronutrients in the gut bacteria [99].

In addition to direct antimicrobial activity, postbiotics could modulate the gut microbiota and inhibit the pathogens, possibly via quorum sensing and adhesion. Quorum sensing is a process of cell-cell communication that allows bacteria to sense population density and regulate their behavior collectively [100]. The block of quorum sensing, called quorum quenching, can be applied in the control of bacterial infections and biofilm formation [101]. Enzymes from bacterial with quorum quenching activity, including lactonases and acylases, showed the ability to degrade the N-Acyl homoserine lactones (AHLs), which led to the inhibition of biofilm formation of *Pseudomonas aeruginosa* PAO1 [102]. Although the approaches targeting quorum sensing were reported as a therapy for pathogens [103], the efficiency and mechanisms of quorum quenching still remain debate which require further investigation [104]. The adhesion ability of probiotics also plays a potential protective role against pathogens through competition for the binding sites in the epithelium [105]. High adherence ability to Caco-2 cells was observed in heat-killed *L. acidophilus* strain LB (*Lactobacillus Boucard*), which exerted the inhibition effect of different diarrheagenic bacteria, including enterotoxigenic and enteropathogenic *E.coli* [106], suggesting the adherence ability still existed in postbiotics.

#### Benefits for gut barrier function

The gut barrier strongly interacts with the gut bacteria, which could regulate the absorption of nutrients, electrolytes, and water from the lumen into the circulation and prevent toxic entities and pathogens [107]. Beneficial effects of postbiotics on the gut barrier were observed by eliminating the risk of intestinal translocation or local inflammation [108]. Pretreatment of SLP from *L. acidophilus* NCFM improved integrity and permeability, restored ZO-1 and occludin protein expression in Caco-2 cells. Moreover, SLP also attenuated the cell apoptosis and inhibited TNF-α by suppressing the activation of NF-κB [109]. Similar protective effects on Caco-2 cells were observed in purified SLPs from *L. plantarum* by increasing the transepithelial resistance and down-regulating permeability [110]. Metabolites such as SCFAs exist in postbiotics could also contribute to the gut barrier function improvement [111]. A study on mice revealed propionate could improve the tight junction through the AKT signaling pathway [112]. Studies showed that administration of acetate, propionate, butyrate alone or in combination boosted transepithelial resistance and stimulated the formation of tight junction in both in vitro and in vivo [113, 114]. Besides, mucin MUC2 expression and secretion can be stimulated by butyrate in goblet cells, which prevents pathogens from destroying enterocytes [115]. Moreover, proteins p40 secreted from LGG could modulate the intestinal epithelial cell homeostasis through the activation of estimated glomerular filtration rate (EGFR) in young adult mouse colon epithelial cells and human colonic epithelial cell line, and T84 cells [116]. An in vitro study showed that protein HM0539 purified from LGG could enhance mucin expression and prevent LPS or TNF-α from inducing gut barrier injury. In mice study, it was verified that HM0539 could promote the development of neonatal intestinal defense and prevent the infection of *E. coli* K1 [117].

#### Immunomodulatory effects on gut

Increasing evidence suggested that substances in postbiotics could interact with the gut immune system and show the potential of immunomodulatory and pharmaceutical effects in individuals [29, 118]. Pattern recognition receptors (PRRs) such as Toll-like receptors (TLRs), nucleotide-binding oligomerization domain-like receptors (NLRs), C-Type lectin-like receptors (CTLRs), and G-protein-coupled receptors (GPCRs) in the gut could recognize components in postbiotics and further induce downstream signaling cascades for beneficial function on the host [65, 119]. The immune functions can be activated by SCFAs through GPCRs, like GPR41, GPR43, and GPR109A, which have shown therapeutic potential in inflammatory bowel diseases [120]. SLP of *L. helveticus* SBT2171 induced the expression of human β-defensin by activating JNK signaling through TLR2 in Caco-2 cells [121]. SLP-8348 from *L. kefiri* increased the expression of IL-6 and IL-10 at both transcription and protein levels, and further improved the murine macrophages’ response to LPS in a Ca^2+^-dependent manner [122]. Furthermore, a study on mice revealed that SLP-8348 exerts immunostimulatory activity through the interactions with mincle [123]. Proteins secreted from probiotics were also observed to have immunomodulatory effects, such as p40 and p75 identified from *Lactobacilli* species [124]. Proteins p40 and p75 produced from LGG can ameliorate the epithelial barrier disruption by a PKC- and MAP kinase-dependent manner [125]. Unmethylated CpG DNA in postbiotics could be recognized by TLR9 and lead to the recruitment of adapter protein MyD88 and activation of NF-κB, which initiate a cascade of innate and adaptive immune responses in the host [126, 127]. Since postbiotics consist of a wide range of molecules, the immunomodulatory effects of postbiotics might not perform by only one single factor. An in vitro study showed expression of prostaglandin E2 and IL-8 was downregulated by CFS of *L. acidophilus*, *L. casei*, *L. lactis*, *L. reuteri*, and *Saccharomyces boulardii* in human colon epithelial HT-29 cells. In addition, peculiar anti-inflammatory effects of supernatants from probiotics were also observed in the modulation of IL-1β, IL-6, TNF-α, and IL-10 production in human macrophages [128]. Heat-killed probiotic bacteria have also been shown to have an immunomodulatory effect in the gut, which are similar to live bacteria [25]. Previous study showed heat-killed lactic acid bacteria such as *L. paracasei* could induce IL-12 secretion that enhances the innate immunity in mice [129]. The addition of heat-inactivated probiotic *B. bifidum* OLB6378 exerts beneficial effects on the mucosal immune system by upregulation of polymeric immunoglobulin receptor mRNA expression in mouse intestinal explant model [130].

### Application of postbiotics in animal production

Apart from the therapeutic effects in mice and human health, postbiotics have been applied in animal production as potential alternatives for antibiotics [131]. We summarized the application of postbiotics in swine, poultry, and ruminants reported in previous studies (Table 1).

**Table 1:** Beneficial effects of different postbiotics supplementation on animal production

Animal	Postbiotics	Dosage	Beneficial effects	Reference
Weaned piglets	HK of *L. rhamnosus*	0.1%, 0.2%, or 0.4% in diet with 1 × 10^9^ CFU/g	Increased growth rate, feed efficiency, and apparent total tract digestibility; decreased TNF-α, TGF-β1, and cortisol in serum	[132, 133]
Weaned piglets	CFS of *L. plantarum* TL1, RS5, RF14, RG11, and RI11	0.3% in diet	Increased feed conversion ratio, lactic acid bacteria count, and SCFA in the gut; decreased fecal pH value, ENT count, and diarrhea incidence	[134]
Weaned piglets	CFS of *L. plantarum* TL1, RS5, RG14	0.5% in diet	Improved average daily gain, feed intake, and protein digestibility; reduce diarrhea incidence, pH value and ENT in the gut	[135]
Weaned piglets	HK of *Enterococcus faecalis* EC-12	0.05% in diet	Improved villous atrophy and increased villous heights in small intestine	[136]
Weaned piglets	HK of *Enterococcus faecium* NHRD IHARA	0.1% in diet with 2 × 10^10^ CFU/kg HK	Improved growth performance, serum IgA and gut morphology; showed same efficacy as live strain	[137, 138]
Weaned piglets	HK of *L. plantarum* L-137	20 mg/kg in diet	Increased *IFN-β* mRNA levels in serum against influenza A virus infection	[139]
Newborn piglets	HK of *L.* spp. and *L. plantarum*	8.6 × 10^7^ CFU/mL	Increased feed intake and weight gain	[140]
Newborn piglets	*L. plantarum* 22F, 25F, *Pediococcus acidilactici* 72 N	1 × 10^9^ CFU/mL	Increased daily gain and feed conversion ratio; increased viable *Lactobacilli* and decreased enterobacterial counts; improve gut morphology	[141]
Broiler chicks at 1-day old	HK of *Enterococcus faecalis* EC-12	0.05% in diet	Increased total IgA in cecal digesta and IgG levels in the serum; reduced VRE colonization in the intestine	[142]
Broiler chicks at 1-day old, layers at 23-week old	CFS of *L. plantarum* TL1, RS5, RF14, RG11, RG14, and RI11	0.3% in diet	Increased in hen-day egg production, reduced fecal pathogens population; increased final body weight, weight gain, feed conversion ratio, gut morphology, and SCFA levels in gut	[143, 144]
Broiler chicks at 1-day old, 22-day old, or 88-day old	CFS of *L. plantarum* RI11	0.3% in diet	Increased body weight, feed conversion ratio; improved villi height in small intestine, increased IgM and IgG levels in serum, increased hepatic *IGF-1* mRNA expression level	[145–148]
Broiler chicks at 14-day old	PC of *Pediococcus acidilactici*, *L. reuteri*, *Enterococcus faecium*, *L. acidophilus*	1 oz/gallon in water	Reduces the proinflammatory response, alternative to antibiotics in the context of *Clostridium perfringens* pathogen challenge	[149]
Broiler chicks at 1-day old	HK of *Bacillus subtilis, L. acidophilus* BFI	2 × 10^8^ CFU/mL	Enhanced feed efficiency, decreased plasma cholesterol and creatinine contents, altered cecal microbiota composition	[150]
Layer hens at 24-week old	HK of *L. salivarius, Bacillus subtilis*	400 g/t in diet	Improved daily egg yield, feed conversion, damaged egg ratio, and Haugh unit; Decreased in total cholesterol, and lipoprotein cholesterol; increased antibody against avian influenza virus	[151]
Postweaning lambs at 112-day old	CFS of *L. plantarum* RG14	0.9% in diet	Increased weight gain, feed intake, nutrient intake, and nutrient digestibility; increased fiber degrading bacteria and decreased total protozoa and methanogens in rumen; lowered leukocyte, lymphocyte, basophil, neutrophil and platelets; improved ruminal epithelium growth and integrity of intestinal barrier; increased IL-6 mRNA and decreased *IL-1β*, *IL-10*, *TNF* mRNA in jejunum	[152–154]

*HK* heat-killed, *CFS* cell-free supernatant, *PC* pure culture, *VRE* vancomycin-resistant enterococci, *SCFA* short chain fatty acid, *ENT* Enterobacteriaceae

#### Swine

Beneficial effects of postbiotics were observed in swine for the growth promoter and regulation of the immune system. Strains from *L. rhamnosus* isolated from pigs were cultured and processed by heating at 80 °C for 30 min. Dietary inclusion 1 × 10^9^ CFU/g of this kind of product could improve production performance, including growth rate, feed efficiency, and apparent total tract digestibility of dry matter in weaned pig. What’s more, pigs fed postbiotics showed reduced post-weaning diarrhea rate together with lower TNF-α, transforming growth factor-β1, and cortisol in serum than that in control group [132, 133]. Feeding of 0.5% metabolites combination from strains of *L. plantarum* TL1, RG14, and RS5 isolated from Malaysian foods in the piglet diet could improve average daily gain and daily feed intake, as well as reduce diarrhea incidence in the postweaning piglets. What’s more, lower Enterobacteriaceae (ENT), higher LAB counts and SCFA levels in the gut of piglets were observed [135]. Similar results were observed in weaned piglets fed with liquid metabolite combinations derived from strains including *L. plantarum* TL1, RG11, RG14, RS5 and RI11. In addition to the improvement of growth performance, metabolite combinations derived from *L. plantarum* strains could contribute to higher villus height of duodenum, suggesting the application of postbiotics could benefit the gut morphology in piglets [134]. Since early weaning usually induced atrophy of villous, oral administration of heat-killed and dried cell preparation of *Enterococcus faecalis* strain EC-12 led to the higher villous of jejunum in piglets weaned at 21-day-old, suggesting the postbiotics could protect the gut health and relief weaning stress in piglets [136]. Also, immunomodulatory ability of postbiotics in weaned piglets was also observed. Oral administration of heat-killed *E. faecium* strain NHRD IHARA led to the increase in serum IgA production in weaned piglets, which showed similar effects with the administration of live cells [137]. On the other hand, heat-killed strain *E. faecium* strain NHRD IHARA also showed beneficial effects on growth performance in pigs [138]. Daily intake of heat-killed *L. plantarum* L-137 induced higher levels of IFN-β and gene expression in the whole blood cells of pigs, which might subsequently augment host defense against the virus infection [139]. However, Busanello et al. showed treatment with inactivated probiotics cells including *L.* spp. and *L. plantarum* showed no significant effects on blood parameters and microbiological counts in the gut of piglets during lactation [140]. Sprat-dried *L. plantarum* strain 22F, 25F, *Pediococcus acidilactici* 72 N isolated from pig feces exhibited beneficial effects in the nursery-finishing pigs, including a better feed conversion ratio, increase of *Lactobacilli* counts, decrease of *Enterobacterial* counts in the gut, which demonstrated the feasibility of substitute for antibiotics [141].

#### Poultry

Postbiotics has been applied in poultry as well. For instance, as mentioned above, heat-killed *Enterococcus faecalis* strain EC-12 also applied in newly hatched broilers from age of 3 to 14. Supplement with heat-killed *Enterococcus faecalis* strain EC-12 increased total IgA in the cecal digesta and total IgG in the serum, and reduced vancomycin-resistant enterococci (VRE) colonization in the intestine, suggesting this kind of postbiotics could stimulate the gut immune system and reinforce the immune reaction against the VRE challenge to accelerate its defecation in chicken [142]. The addition of metabolite combination of *L. plantarum* RS5, RI11, RG14, and RG11 strains could increase fecal lactic acid bacteria counts, villus height, and volatile fatty acids in broiler chickens [143]. What’s more, chicks fed with CFS of *L. plantarum* RI11 showed improvement of growth performance, including higher final body weight, total weight gain and average daily gain than other groups, suggesting the *L. plantarum* RI11 could be used as an alternative antibiotic growth promoter. Also, supplementation of postbiotics improved the gut morphology, lowered ENT and *E. coli* counts and caecal pH value in the gut but showed limited effect on plasma IgA level [145, 146]. Anti-stress effects of postbiotics *L. plantarum* RI11 were also observed via regulation of antioxidant enzyme activity, gut barrier genes, and cytokine, acute phase proteins in broilers [147, 148]. In addition, postbiotic metabolite combinations derived from *L. plantarum* strains RI11 also reduced fecal ENT levels, improved egg quality and increase hen-day egg production in laying hens [144]. Apart from strains from *Lactobacillus*, postbiotics from *Bacillus subtilis* also showed beneficial effects in laying hens and broilers, including feed efficiency, egg quality, and immune response [150, 151]. Postbiotics product from a cocktail containing *Pediococcus acidilactici*, *L. reuteri*, *Enterococcus faecium*, and *L. acidophilus* could improve weight gain and alleviate the proinflammatory responses after the challenge of *Clostridium perfringens* in broilers [149].

#### Ruminants

In ruminants, an in vitro study revealed the alteration of rumen fermentation and bacteria composition after supplementary of postbiotics from *L. plantarum* RG14, including elevated ruminal volatile fatty acid (VFA) and population of total bacteria, cellulolytic bacteria, and total protozoa [155]. When the same postbiotics were applied in postweaning lambs for 60 d, improvement of rumen epithelium and intestinal barrier function was observed, including the increase of ruminal papillae growth and upregulation of tight junction protein-1, Claudin-1, and Claudin-4 mRNA levels. Lambs fed with postbiotics from *L. plantarum* RG14 also showed increase of *IL-6* mRNA and decrease of mRNA of *IL-1β*, *IL-10*, *TNF* in the jejunum, suggesting the immunomodulation effects of postbiotics in ruminants [152–154].

## Conclusions

The utilization of postbiotics has shown great potential and can be an alternative to antibiotics in animal production (Fig. 1). However, despite the fact that the inanimate of postbiotics makes it more stable and safer than probiotics, the exact composition in postbiotics remains to be identified in the future, which would make it more capable and convinced in the application. Moreover, although studies have investigated the mechanism of a single factor in postbiotics, the complex interaction between diverse compounds and host can exist. Therefore, as an integration of various compounds, the exact mechanism of the postbiotics is needed to be further illustrated in future studies.

## Data Availability

Not applicable.

## Abbreviations

AHLs: N-Acyl homoserine lactones
CpG motifs: Cytosine-guanine dinucleotide in particular base contexts
CPS: Capsular polysaccharides
CTLRs: C-Type lectin-like receptors
EGFR: Estimated glomerular filtration rate
EPS: Exopolysaccharides
GPCRs: G-protein-coupled receptors
ISAPP: International Scientific Association of Probiotics and Prebiotics
JNK: c-Jun N-terminal kinase
LAB: Lactic acid bacteria
LGG: *Lactobacillus rhamnosus* GG
LTAs: Lipoteichoic acids
NLR: Nucleotide-binding oligomerization domain-like receptors
ONDs: Oligodeoxynucleotides
PRRs: Pattern recognition receptors
SCFAs: Short-chain fatty acids
SLP: Surface layer proteins
TEER: Transepithelial electrical resistance
TLR: Toll-like receptor
WPS: Wall polysaccharides
WTAs: Wall teichoic acids
ENT: *Enterobacteriaceae*
VRE: Vancomycin-resistant *Enterococci*
